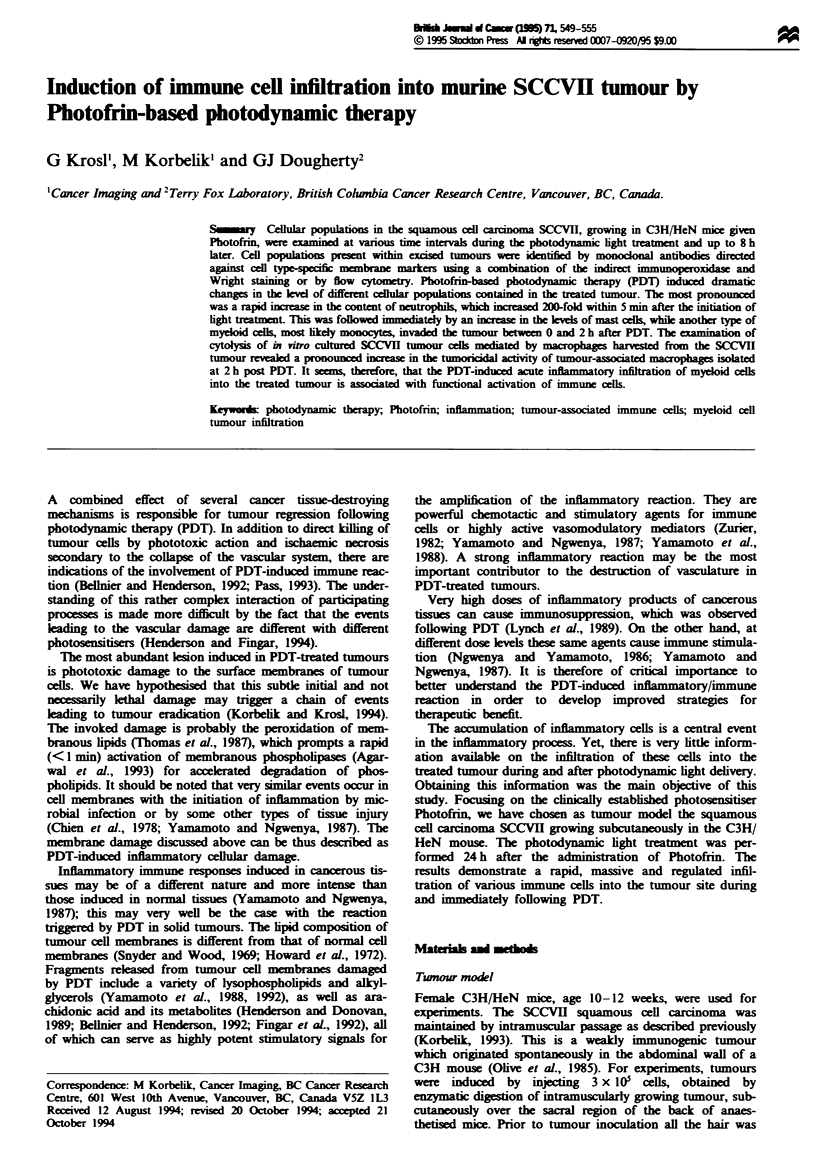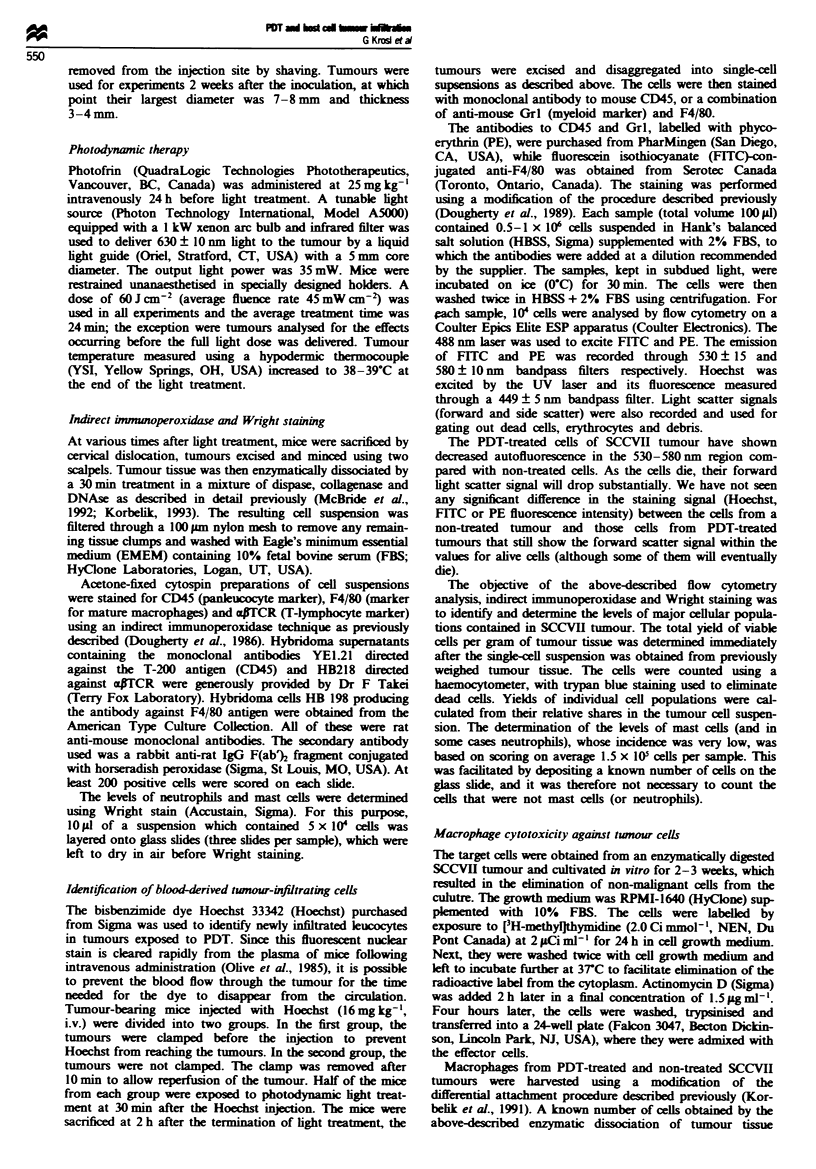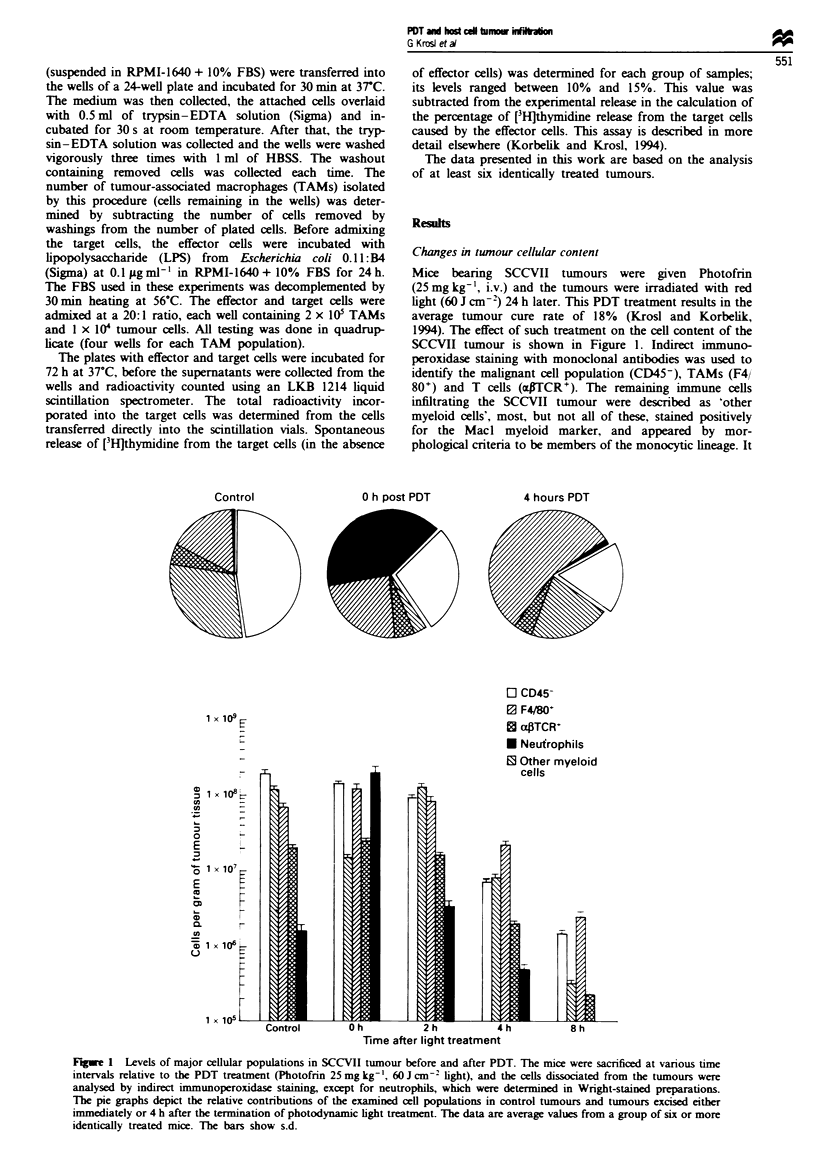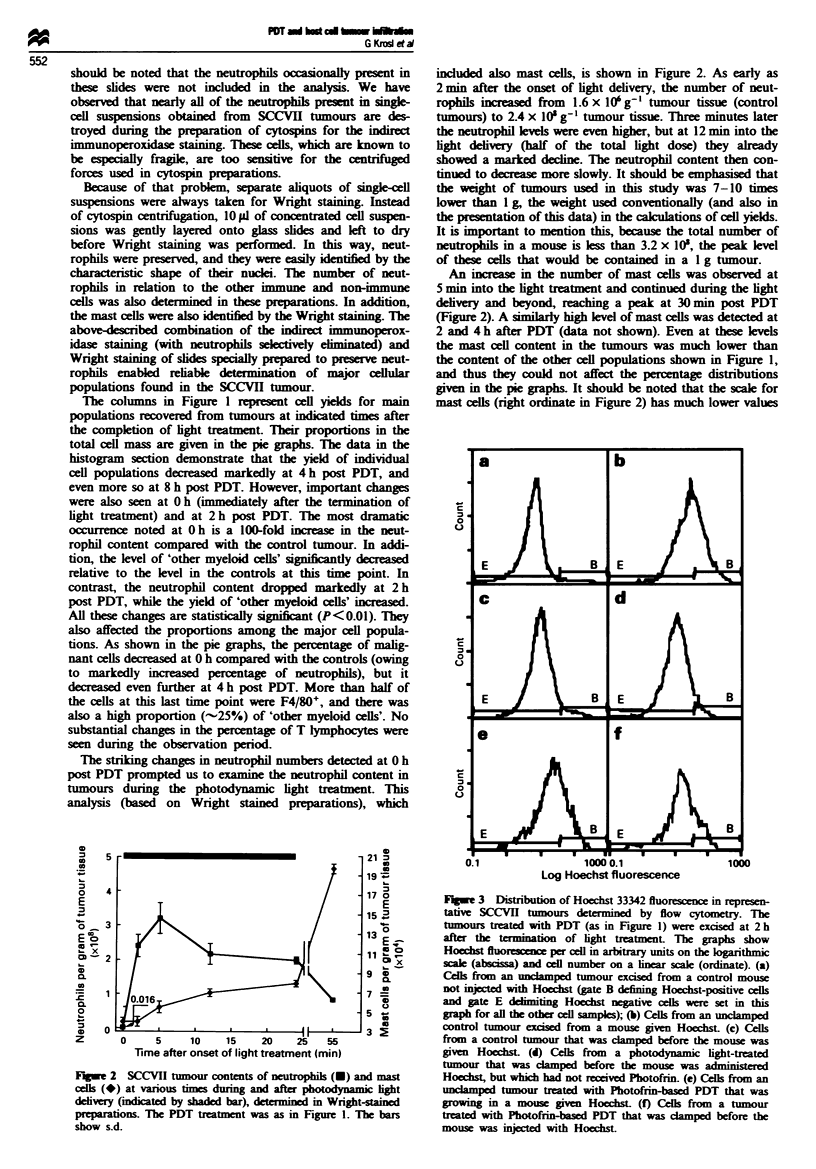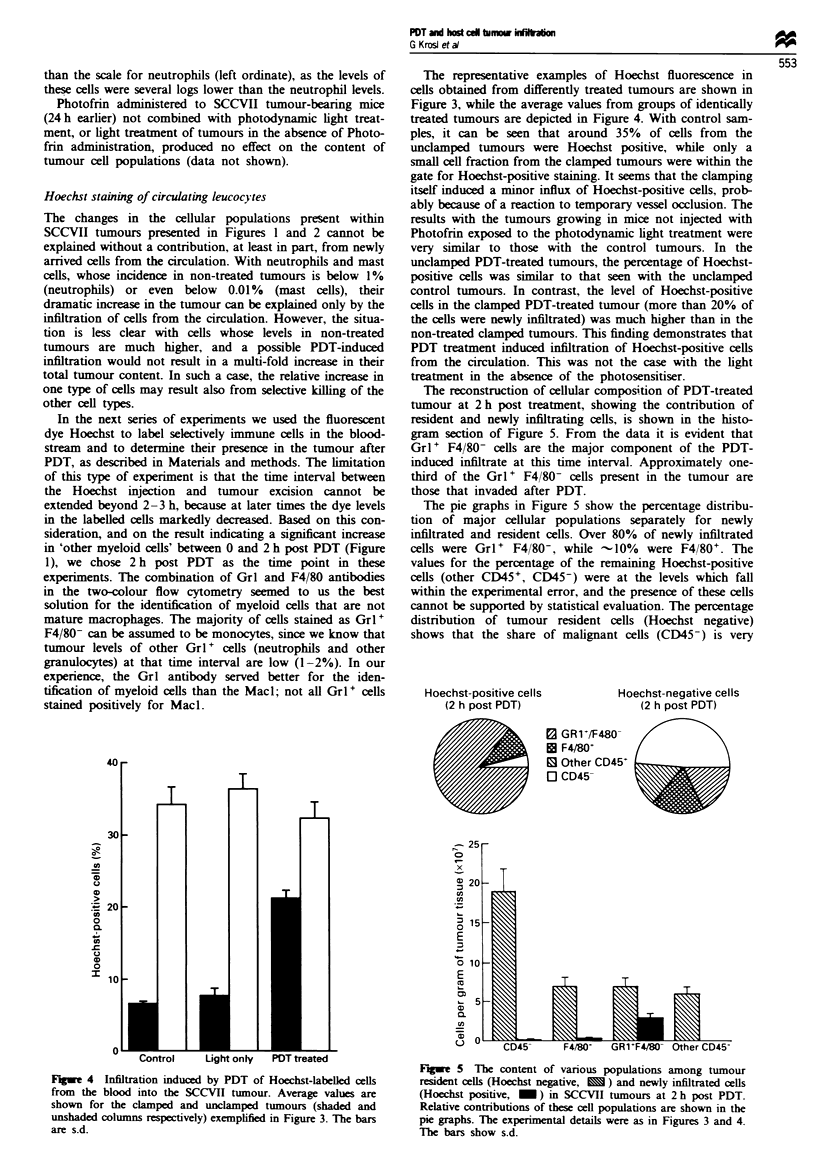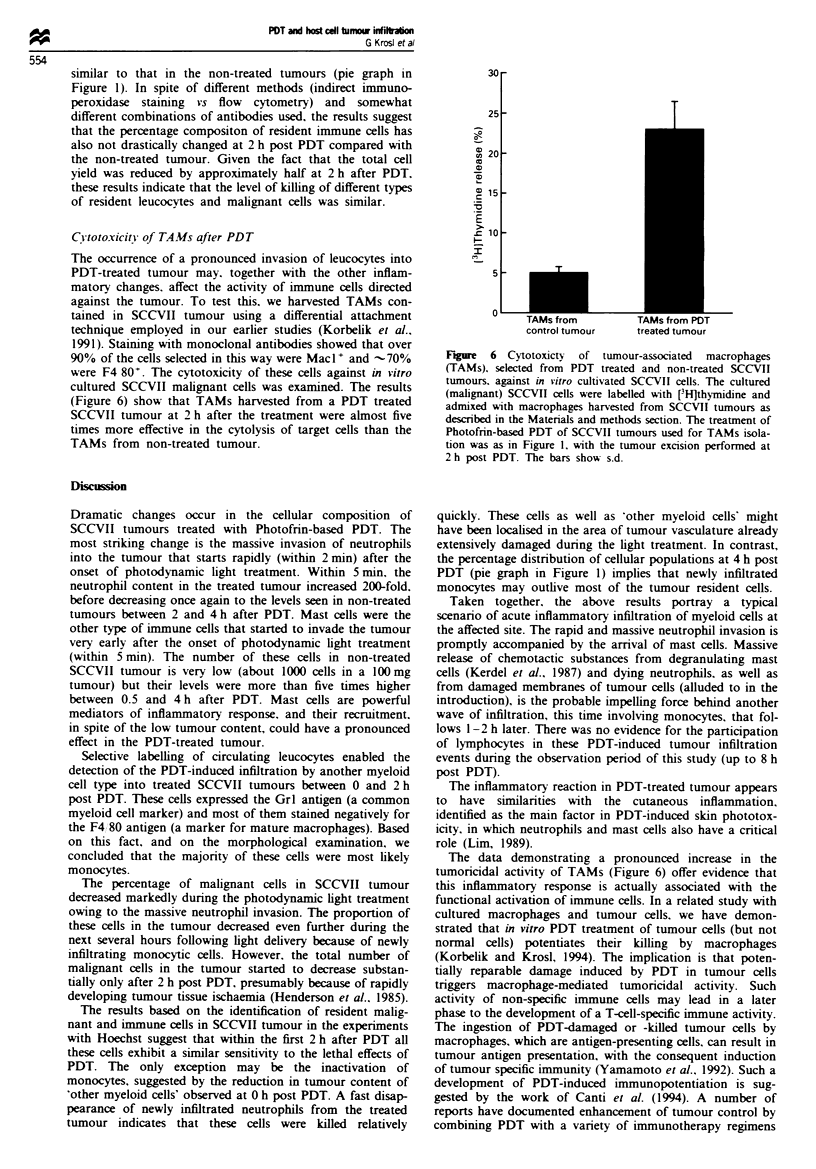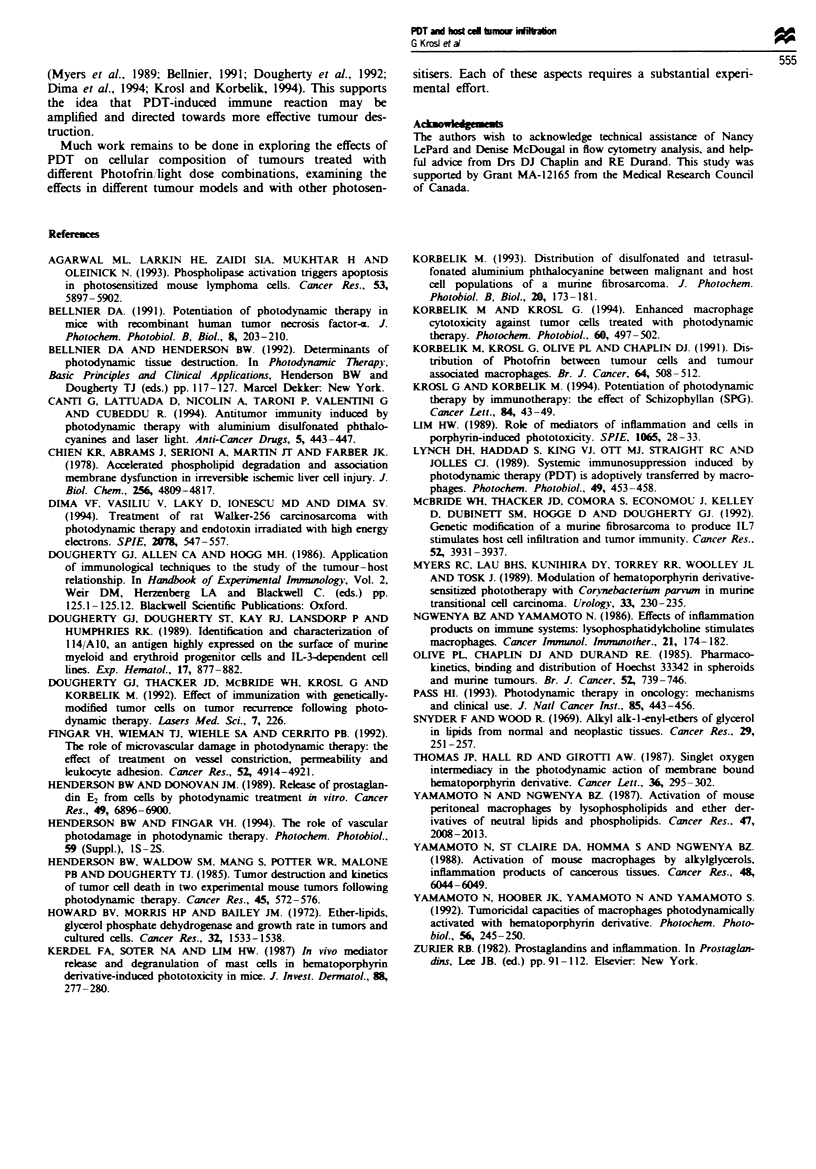# Induction of immune cell infiltration into murine SCCVII tumour by photofrin-based photodynamic therapy.

**DOI:** 10.1038/bjc.1995.108

**Published:** 1995-03

**Authors:** G. Krosl, M. Korbelik, G. J. Dougherty

**Affiliations:** Cancer Imaging, British Columbia Cancer Research Centre, Vancouver, Canada.

## Abstract

Cellular populations in the squamous cell carcinoma SCCVII, growing in C3H/HeN mice given Photofrin, were examined at various time intervals during the photodynamic light treatment and up to 8 h later. Cell populations present within excised tumours were identified by monoclonal antibodies directed against cell type-specific membrane markers using a combination of the indirect immunoperoxidase and Wright staining or by flow cytometry. Photofrin-based photodynamic therapy (PDT) induced dramatic changes in the level of different cellular populations contained in the treated tumour. The most pronounced was a rapid increase in the content of neutrophils, which increased 200-fold within 5 min after the initiation of light treatment. This was followed immediately by an increase in the levels of mast cells, while another type of myeloid cells, most likely monocytes, invaded the tumour between 0 and 2 h after PDT. The examination of cytolysis of in vitro cultured SCCVII tumour cells mediated by macrophages harvested from the SCCVII tumour revealed a pronounced increase in the tumoricidal activity of tumour-associated macrophages isolated at 2 h post PDT. It seems, therefore, that the PDT-induced acute inflammatory infiltration of myeloid cells into the treated tumour is associated with functional activation of immune cells.


© 1995 Stockton Press All rights reserved 0007-0920/95 $9.00

### Induction of immune cell infiltration into murine SCCVII tumour by Photofrin-based photodynamic therapy

G Krosl[1], M Korbelik[1] and GJ Dougherty[2]

[1]*Cancer Imaging and* [2]*Terry Fox Laboratory, British Columbia Cancer Research Centre, Vancouver, BC, Canada.*

**Summary** Cellular populations in the squamous cell carcinoma SCCVII, growing in C3H/HeN mice given Photofrin, were examined at various time intervals during the photodynamic light treatment and up to 8 h later. Cell populations present within excised tumours were identified by monoclonal antibodies directed against cell type-specific membrane markers using a combination of the indirect immunoperoxidase and Wright staining or by flow cytometry. Photofrin-based photodynamic therapy (PDT) induced dramatic changes in the level of different cellular populations contained in the treated tumour. The most pronounced was a rapid increase in the content of neutrophils, which increased 200-fold within 5 min after the initiation of light treatment. This was followed immediately by an increase in the levels of mast cells, while another type of myeloid cells, most likely monocytes, invaded the tumour between 0 and 2 h after PDT. The examination of cytolysis of *in vitro* cultured SCCVII tumour cells mediated by macrophages harvested from the SCCVII tumour revealed a pronounced increase in the tumoricidal activity of tumour-associated macrophages isolated at 2 h post PDT. It seems, therefore, that the PDT-induced acute inflammatory infiltration of myeloid cells into the treated tumour is associated with functional activation of immune cells.

**Keywords:** photodynamic therapy; Photofrin; inflammation; tumour-associated immune cells; myeloid cell tumour infiltration

A combined effect of several cancer tissue-destroying mechanisms is responsible for tumour regression following photodynamic therapy (PDT). In addition to direct killing of tumour cells by phototoxic action and ischaemic necrosis secondary to the collapse of the vascular system, there are indications of the involvement of PDT-induced immune reaction (Bellnier and Henderson, 1992; Pass, 1993). The understanding of this rather complex interaction of participating processes is made more difficult by the fact that the events leading to the vascular damage are different with different photosensitisers (Henderson and Fingar, 1994).

The most abundant lesion induced in PDT-treated tumours is phototoxic damage to the surface membranes of tumour cells. We have hypothesised that this subtle initial and not necessarily lethal damage may trigger a chain of events leading to tumour eradication (Korbelik and Krosl, 1994). The invoked damage is probably the peroxidation of membranous lipids (Thomas *et al.*, 1987), which prompts a rapid (<1 min) activation of membranous phospholipases (Agarwal *et al.*, 1993) for accelerated degradation of phospholipids. It should be noted that very similar events occur in cell membranes with the initiation of inflammation by microbial infection or by some other types of tissue injury (Chien *et al.*, 1978; Yamamoto and Ngwenya, 1987). The membrane damage discussed above can be thus described as PDT-induced inflammatory cellular damage.

Inflammatory immune responses induced in cancerous tissues may be of a different nature and more intense than those induced in normal tissues (Yamamoto and Ngwenya, 1987); this may very well be the case with the reaction triggered by PDT in solid tumours. The lipid composition of tumour cell membranes is different from that of normal cell membranes (Snyder and Wood, 1969; Howard *et al.*, 1972). Fragments released from tumour cell membranes damaged by PDT include a variety of lysophospholipids and alkylglycerols (Yamamoto *et al.*, 1988, 1992), as well as arachidonic acid and its metabolites (Henderson and Donovan, 1989; Bellnier and Henderson, 1992; Fingar *et al.*, 1992), all of which can serve as highly potent stimulatory signals for the amplification of the inflammatory reaction. They are powerful chemotactic and stimulatory agents for immune cells or highly active vasomodulatory mediators (Zurier, 1982; Yamamoto and Ngwenya, 1987; Yamamoto *et al.*, 1988). A strong inflammatory reaction may be the most important contributor to the destruction of vasculature in PDT-treated tumours.

Very high doses of inflammatory products of cancerous tissues can cause immunosuppression, which was observed following PDT (Lynch *et al.*, 1989). On the other hand, at different dose levels these same agents cause immune stimulation (Ngwenya and Yamamoto, 1986; Yamamoto and Ngwenya, 1987). It is therefore of critical importance to better understand the PDT-induced inflammatory/immune reaction in order to develop improved strategies for therapeutic benefit.

The accumulation of inflammatory cells is a central event in the inflammatory process. Yet, there is very little information available on the infiltration of these cells into the treated tumour during and after photodynamic light delivery. Obtaining this information was the main objective of this study. Focusing on the clinically established photosensitiser Photofrin, we have chosen as tumour model the squamous cell carcinoma SCCVII growing subcutaneously in the C3H/HeN mouse. The photodynamic light treatment was performed 24 h after the administration of Photofrin. The results demonstrate a rapid, massive and regulated infiltration of various immune cells into the tumour site during and immediately following PDT.

### Materials and methods

### *Tumour model*

Female C3H/HeN mice, age 10-12 weeks, were used for experiments. The SCCVII squamous cell carcinoma was maintained by intramuscular passage as described previously (Korbelik, 1993). This is a weakly immunogenic tumour which originated spontaneously in the abdominal wall of a C3H mouse (Olive *et al.*, 1985). For experiments, tumours were induced by injecting $3 \times 10^5$ cells, obtained by enzymatic digestion of intramuscularly growing tumour, subcutaneously over the sacral region of the back of anaesthetised mice. Prior to tumour inoculation all the hair was

Correspondence: M Korbelik, Cancer Imaging, BC Cancer Research Centre, 601 West 10th Avenue, Vancouver, BC, Canada V5Z 1L3
Received 12 August 1994; revised 20 October 1994; accepted 21 October 1994

removed from the injection site by shaving. Tumours were used for experiments 2 weeks after the inoculation, at which point their largest diameter was 7-8 mm and thickness 3-4 mm.

*Photodynamic therapy*

Photofrin (QuadraLogic Technologies Phototherapeutics, Vancouver, BC, Canada) was administered at 25 mg $kg^{-1}$ intravenously 24 h before light treatment. A tunable light source (Photon Technology International, Model A5000) equipped with a 1 kW xenon arc bulb and infrared filter was used to deliver 630 ± 10 nm light to the tumour by a liquid light guide (Oriel, Stratford, CT, USA) with a 5 mm core diameter. The output light power was 35 mW. Mice were restrained unanaesthetised in specially designed holders. A dose of 60 J $cm^{-2}$ (average fluence rate 45 mW $cm^{-2}$) was used in all experiments and the average treatment time was 24 min; the exception were tumours analysed for the effects occurring before the full light dose was delivered. Tumour temperature measured using a hypodermic thermocouple (YSI, Yellow Springs, OH, USA) increased to 38-39°C at the end of the light treatment.

*Indirect immunoperoxidase and Wright staining*

At various times after light treatment, mice were sacrificed by cervical dislocation, tumours excised and minced using two scalpels. Tumour tissue was then enzymatically dissociated by a 30 min treatment in a mixture of dispase, collagenase and DNAse as described in detail previously (McBride *et al.*, 1992; Korbelik, 1993). The resulting cell suspension was filtered through a 100 μm nylon mesh to remove any remaining tissue clumps and washed with Eagle's minimum essential medium (EMEM) containing 10% fetal bovine serum (FBS; HyClone Laboratories, Logan, UT, USA).

Acetone-fixed cytospin preparations of cell suspensions were stained for CD45 (panleucocyte marker), F4/80 (marker for mature macrophages) and αβTCR (T-lymphocyte marker) using an indirect immunoperoxidase technique as previously described (Dougherty *et al.*, 1986). Hybridoma supernatants containing the monoclonal antibodies YE1.21 directed against the T-200 antigen (CD45) and HB218 directed against αβTCR were generously provided by Dr F Takei (Terry Fox Laboratory). Hybridoma cells HB 198 producing the antibody against F4/80 antigen were obtained from the American Type Culture Collection. All of these were rat anti-mouse monoclonal antibodies. The secondary antibody used was a rabbit anti-rat IgG $F(ab')_2$ fragment conjugated with horseradish peroxidase (Sigma, St Louis, MO, USA). At least 200 positive cells were scored on each slide.

The levels of neutrophils and mast cells were determined using Wright stain (Accustain, Sigma). For this purpose, 10 μl of a suspension which contained $5 \times 10^4$ cells was layered onto glass slides (three slides per sample), which were left to dry in air before Wright staining.

*Identification of blood-derived tumour-infiltrating cells*

The bisbenzimide dye Hoechst 33342 (Hoechst) purchased from Sigma was used to identify newly infiltrated leucocytes in tumours exposed to PDT. Since this fluorescent nuclear stain is cleared rapidly from the plasma of mice following intravenous administration (Olive *et al.*, 1985), it is possible to prevent the blood flow through the tumour for the time needed for the dye to disappear from the circulation. Tumour-bearing mice injected with Hoechst (16 mg $kg^{-1}$, i.v.) were divided into two groups. In the first group, the tumours were clamped before the injection to prevent Hoechst from reaching the tumours. In the second group, the tumours were not clamped. The clamp was removed after 10 min to allow reperfusion of the tumour. Half of the mice from each group were exposed to photodynamic light treatment at 30 min after the Hoechst injection. The mice were sacrificed at 2 h after the termination of light treatment, the tumours were excised and disaggregated into single-cell suspensions as described above. The cells were then stained with monoclonal antibody to mouse CD45, or a combination of anti-mouse Gr1 (myeloid marker) and F4/80.

The antibodies to CD45 and Gr1, labelled with phycoerythrin (PE), were purchased from PharMingen (San Diego, CA, USA), while fluorescein isothiocyanate (FITC)-conjugated anti-F4/80 was obtained from Serotec Canada (Toronto, Ontario, Canada). The staining was performed using a modification of the procedure described previously (Dougherty *et al.*, 1989). Each sample (total volume 100 μl) contained $0.5-1 \times 10^6$ cells suspended in Hank's balanced salt solution (HBSS, Sigma) supplemented with 2% FBS, to which the antibodies were added at a dilution recommended by the supplier. The samples, kept in subdued light, were incubated on ice (0°C) for 30 min. The cells were then washed twice in HBSS + 2% FBS using centrifugation. For each sample, $10^4$ cells were analysed by flow cytometry on a Coulter Epics Elite ESP apparatus (Coulter Electronics). The 488 nm laser was used to excite FITC and PE. The emission of FITC and PE was recorded through 530 ± 15 and 580 ± 10 nm bandpass filters respectively. Hoechst was excited by the UV laser and its fluorescence measured through a 449 ± 5 nm bandpass filter. Light scatter signals (forward and side scatter) were also recorded and used for gating out dead cells, erythrocytes and debris.

The PDT-treated cells of SCCVII tumour have shown decreased autofluorescence in the 530-580 nm region compared with non-treated cells. As the cells die, their forward light scatter signal will drop substantially. We have not seen any significant difference in the staining signal (Hoechst, FITC or PE fluorescence intensity) between the cells from a non-treated tumour and those cells from PDT-treated tumours that still show the forward scatter signal within the values for alive cells (although some of them will eventually die).

The objective of the above-described flow cytometry analysis, indirect immunoperoxidase and Wright staining was to identify and determine the levels of major cellular populations contained in SCCVII tumour. The total yield of viable cells per gram of tumour tissue was determined immediately after the single-cell suspension was obtained from previously weighed tumour tissue. The cells were counted using a haemocytometer, with trypan blue staining used to eliminate dead cells. Yields of individual cell populations were calculated from their relative shares in the tumour cell suspension. The determination of the levels of mast cells (and in some cases neutrophils), whose incidence was very low, was based on scoring on average $1.5 \times 10^5$ cells per sample. This was facilitated by depositing a known number of cells on the glass slide, and it was therefore not necessary to count the cells that were not mast cells (or neutrophils).

*Macrophage cytotoxicity against tumour cells*

The target cells were obtained from an enzymatically digested SCCVII tumour and cultivated *in vitro* for 2-3 weeks, which resulted in the elimination of non-malignant cells from the culutre. The growth medium was RPMI-1640 (HyClone) supplemented with 10% FBS. The cells were labelled by exposure to [$^3$H-methyl]thymidine (2.0 Ci $mmol^{-1}$, NEN, Du Pont Canada) at 2 μCi $ml^{-1}$ for 24 h in cell growth medium. Next, they were washed twice with cell growth medium and left to incubate further at 37°C to facilitate elimination of the radioactive label from the cytoplasm. Actinomycin D (Sigma) was added 2 h later in a final concentration of 1.5 μg $ml^{-1}$. Four hours later, the cells were washed, trypsinised and transferred into a 24-well plate (Falcon 3047, Becton Dickinson, Lincoln Park, NJ, USA), where they were admixed with the effector cells.

Macrophages from PDT-treated and non-treated SCCVII tumours were harvested using a modification of the differential attachment procedure described previously (Korbelik *et al.*, 1991). A known number of cells obtained by the above-described enzymatic dissociation of tumour tissue

(suspended in RPMI-1640 + 10% FBS) were transferred into the wells of a 24-well plate and incubated for 30 min at 37°C. The medium was then collected, the attached cells overlaid with 0.5 ml of trypsin-EDTA solution (Sigma) and incubated for 30 s at room temperature. After that, the trypsin-EDTA solution was collected and the wells were washed vigorously three times with 1 ml of HBSS. The washout containing removed cells was collected each time. The number of tumour-associated macrophages (TAMs) isolated by this procedure (cells remaining in the wells) was determined by subtracting the number of cells removed by washings from the number of plated cells. Before admixing the target cells, the effector cells were incubated with lipopolysaccharide (LPS) from *Escherichia coli* 0.11:B4 (Sigma) at 0.1 $\mu g\ ml^{-1}$ in RPMI-1640 + 10% FBS for 24 h. The FBS used in these experiments was decomplemented by 30 min heating at 56°C. The effector and target cells were admixed at a 20:1 ratio, each well containing $2 \times 10^5$ TAMs and $1 \times 10^4$ tumour cells. All testing was done in quadruplicate (four wells for each TAM population).

The plates with effector and target cells were incubated for 72 h at 37°C, before the supernatants were collected from the wells and radioactivity counted using an LKB 1214 liquid scintillation spectrometer. The total radioactivity incorporated into the target cells was determined from the cells transferred directly into the scintillation vials. Spontaneous release of [$^3$H]thymidine from the target cells (in the absence of effector cells) was determined for each group of samples; its levels ranged between 10% and 15%. This value was subtracted from the experimental release in the calculation of the percentage of [$^3$H]thymidine release from the target cells caused by the effector cells. This assay is described in more detail elsewhere (Korbelik and Krosl, 1994).

The data presented in this work are based on the analysis of at least six identically treated tumours.

### Results

### *Changes in tumour cellular content*

Mice bearing SCCVII tumours were given Photofrin (25 mg $kg^{-1}$, i.v.) and the tumours were irradiated with red light (60 J $cm^{-2}$) 24 h later. This PDT treatment results in the average tumour cure rate of 18% (Krosl and Korbelik, 1994). The effect of such treatment on the cell content of the SCCVII tumour is shown in Figure 1. Indirect immunoperoxidase staining with monoclonal antibodies was used to identify the malignant cell population (CD45$^-$), TAMs (F4/80$^+$) and T cells ($\alpha\beta$TCR$^+$). The remaining immune cells infiltrating the SCCVII tumour were described as 'other myeloid cells', most, but not all of these, stained positively for the Mac1 myeloid marker, and appeared by morphological criteria to be members of the monocytic lineage. It

**Figure 1** Levels of major cellular populations in SCCVII tumour before and after PDT. The mice were sacrificed at various time intervals relative to the PDT treatment (Photofrin 25 mg $kg^{-1}$, 60 J $cm^{-2}$ light), and the cells dissociated from the tumours were analysed by indirect immunoperoxidase staining, except for neutrophils, which were determined in Wright-stained preparations. The pie graphs depict the relative contributions of the examined cell populations in control tumours and tumours excised either immediately or 4 h after the termination of photodynamic light treatment. The data are average values from a group of six or more identically treated mice. The bars show s.d.

should be noted that the neutrophils occasionally present in these slides were not included in the analysis. We have observed that nearly all of the neutrophils present in single-cell suspensions obtained from SCCVII tumours are destroyed during the preparation of cytospins for the indirect immunoperoxidase staining. These cells, which are known to be especially fragile, are too sensitive for the centrifuged forces used in cytospin preparations.

Because of that problem, separate aliquots of single-cell suspensions were always taken for Wright staining. Instead of cytospin centrifugation, 10 µl of concentrated cell suspensions was gently layered onto glass slides and left to dry before Wright staining was performed. In this way, neutrophils were preserved, and they were easily identified by the characteristic shape of their nuclei. The number of neutrophils in relation to the other immune and non-immune cells was also determined in these preparations. In addition, the mast cells were also identified by the Wright staining. The above-described combination of the indirect immunoperoxidase staining (with neutrophils selectively eliminated) and Wright staining of slides specially prepared to preserve neutrophils enabled reliable determination of major cellular populations found in the SCCVII tumour.

The columns in Figure 1 represent cell yields for main populations recovered from tumours at indicated times after the completion of light treatment. Their proportions in the total cell mass are given in the pie graphs. The data in the histogram section demonstrate that the yield of individual cell populations decreased markedly at 4 h post PDT, and even more so at 8 h post PDT. However, important changes were also seen at 0 h (immediately after the termination of light treatment) and at 2 h post PDT. The most dramatic occurrence noted at 0 h is a 100-fold increase in the neutrophil content compared with the control tumour. In addition, the level of 'other myeloid cells' significantly decreased relative to the level in the controls at this time point. In contrast, the neutrophil content dropped markedly at 2 h post PDT, while the yield of 'other myeloid cells' increased. All these changes are statistically significant ($P<0.01$). They also affected the proportions among the major cell populations. As shown in the pie graphs, the percentage of malignant cells decreased at 0 h compared with the controls (owing to markedly increased percentage of neutrophils), but it decreased even further at 4 h post PDT. More than half of the cells at this last time point were F4/80$^+$, and there was also a high proportion (~25%) of 'other myeloid cells'. No substantial changes in the percentage of T lymphocytes were seen during the observation period.

The striking changes in neutrophil numbers detected at 0 h post PDT prompted us to examine the neutrophil content in tumours during the photodynamic light treatment. This analysis (based on Wright stained preparations), which included also mast cells, is shown in Figure 2. As early as 2 min after the onset of light delivery, the number of neutrophils increased from $1.6 \times 10^6$ g$^{-1}$ tumour tissue (control tumours) to $2.4 \times 10^8$ g$^{-1}$ tumour tissue. Three minutes later the neutrophil levels were even higher, but at 12 min into the light delivery (half of the total light dose) they already showed a marked decline. The neutrophil content then continued to decrease more slowly. It should be emphasised that the weight of tumours used in this study was 7–10 times lower than 1 g, the weight used conventionally (and also in the presentation of this data) in the calculations of cell yields. It is important to mention this, because the total number of neutrophils in a mouse is less than $3.2 \times 10^8$, the peak level of these cells that would be contained in a 1 g tumour.

An increase in the number of mast cells was observed at 5 min into the light treatment and continued during the light delivery and beyond, reaching a peak at 30 min post PDT (Figure 2). A similarly high level of mast cells was detected at 2 and 4 h after PDT (data not shown). Even at these levels the mast cell content in the tumours was much lower than the content of the other cell populations shown in Figure 1, and thus they could not affect the percentage distributions given in the pie graphs. It should be noted that the scale for mast cells (right ordinate in Figure 2) has much lower values

**Figure 2** SCCVII tumour contents of neutrophils (■) and mast cells (◆) at various times during and after photodynamic light delivery (indicated by shaded bar), determined in Wright-stained preparations. The PDT treatment was as in Figure 1. The bars show s.d.

**Figure 3** Distribution of Hoechst 33342 fluorescence in representative SCCVII tumours determined by flow cytometry. The tumours treated with PDT (as in Figure 1) were excised at 2 h after the termination of light treatment. The graphs show Hoechst fluorescence per cell in arbitrary units on the logarithmic scale (abscissa) and cell number on a linear scale (ordinate). (**a**) Cells from an unclamped tumour excised from a control mouse not injected with Hoechst (gate B defining Hoechst-positive cells and gate E delimiting Hoechst negative cells were set in this graph for all the other cell samples); (**b**) Cells from an unclamped control tumour excised from a mouse given Hoechst. (**c**) Cells from a control tumour that was clamped before the mouse was given Hoechst. (**d**) Cells from a photodynamic light-treated tumour that was clamped before the mouse was administered Hoechst, but which had not received Photofrin. (**e**) Cells from an unclamped tumour treated with Photofrin-based PDT that was growing in a mouse given Hoechst. (**f**) Cells from a tumour treated with Photofrin-based PDT that was clamped before the mouse was injected with Hoechst.

than the scale for neutrophils (left ordinate), as the levels of these cells were several logs lower than the neutrophil levels.

Photofrin administered to SCCVII tumour-bearing mice (24 h earlier) not combined with photodynamic light treatment, or light treatment of tumours in the absence of Photofrin administration, produced no effect on the content of tumour cell populations (data not shown).

*Hoechst staining of circulating leucocytes*

The changes in the cellular populations present within SCCVII tumours presented in Figures 1 and 2 cannot be explained without a contribution, at least in part, from newly arrived cells from the circulation. With neutrophils and mast cells, whose incidence in non-treated tumours is below 1% (neutrophils) or even below 0.01% (mast cells), their dramatic increase in the tumour can be explained only by the infiltration of cells from the circulation. However, the situation is less clear with cells whose levels in non-treated tumours are much higher, and a possible PDT-induced infiltration would not result in a multi-fold increase in their total tumour content. In such a case, the relative increase in one type of cells may result also from selective killing of the other cell types.

In the next series of experiments we used the fluorescent dye Hoechst to label selectively immune cells in the bloodstream and to determine their presence in the tumour after PDT, as described in Materials and methods. The limitation of this type of experiment is that the time interval between the Hoechst injection and tumour excision cannot be extended beyond 2–3 h, because at later times the dye levels in the labelled cells markedly decreased. Based on this consideration, and on the result indicating a significant increase in 'other myeloid cells' between 0 and 2 h post PDT (Figure 1), we chose 2 h post PDT as the time point in these experiments. The combination of Grl and F4/80 antibodies in the two-colour flow cytometry seemed to us the best solution for the identification of myeloid cells that are not mature macrophages. The majority of cells stained as $Grl^+$ $F4/80^-$ can be assumed to be monocytes, since we know that tumour levels of other $Grl^+$ cells (neutrophils and other granulocytes) at that time interval are low (1–2%). In our experience, the Grl antibody served better for the identification of myeloid cells than the Macl; not all $Grl^+$ cells stained positively for Macl.

**Figure 4** Infiltration induced by PDT of Hoechst-labelled cells from the blood into the SCCVII tumour. Average values are shown for the clamped and unclamped tumours (shaded and unshaded columns respectively) exemplified in Figure 3. The bars are s.d.

The representative examples of Hoechst fluorescence in cells obtained from differently treated tumours are shown in Figure 3, while the average values from groups of identically treated tumours are depicted in Figure 4. With control samples, it can be seen that around 35% of cells from the unclamped tumours were Hoechst positive, while only a small cell fraction from the clamped tumours were within the gate for Hoechst-positive staining. It seems that the clamping itself induced a minor influx of Hoechst-positive cells, probably because of a reaction to temporary vessel occlusion. The results with the tumours growing in mice not injected with Photofrin exposed to the photodynamic light treatment were very similar to those with the control tumours. In the unclamped PDT-treated tumours, the percentage of Hoechst-positive cells was similar to that seen with the unclamped control tumours. In contrast, the level of Hoechst-positive cells in the clamped PDT-treated tumour (more than 20% of the cells were newly infiltrated) was much higher than in the non-treated clamped tumours. This finding demonstrates that PDT treatment induced infiltration of Hoechst-positive cells from the circulation. This was not the case with the light treatment in the absence of the photosensitiser.

The reconstruction of cellular composition of PDT-treated tumour at 2 h post treatment, showing the contribution of resident and newly infiltrating cells, is shown in the histogram section of Figure 5. From the data it is evident that $Grl^+$ $F4/80^-$ cells are the major component of the PDT-induced infiltrate at this time interval. Approximately one-third of the $Grl^+$ $F4/80^-$ cells present in the tumour are those that invaded after PDT.

The pie graphs in Figure 5 show the percentage distribution of major cellular populations separately for newly infiltrated and resident cells. Over 80% of newly infiltrated cells were $Grl^+$ $F4/80^-$, while ~10% were $F4/80^+$. The values for the percentage of the remaining Hoechst-positive cells (other $CD45^+$, $CD45^-$) were at the levels which fall within the experimental error, and the presence of these cells cannot be supported by statistical evaluation. The percentage distribution of tumour resident cells (Hoechst negative) shows that the share of malignant cells ($CD45^-$) is very

**Figure 5** The content of various populations among tumour resident cells (Hoechst negative, ▧) and newly infiltrated cells (Hoechst positive, ■) in SCCVII tumours at 2 h post PDT. Relative contributions of these cell populations are shown in the pie graphs. The experimental details were as in Figures 3 and 4. The bars show s.d.

similar to that in the non-treated tumours (pie graph in Figure 1). In spite of different methods (indirect immunoperoxidase staining *vs* flow cytometry) and somewhat different combinations of antibodies used, the results suggest that the percentage compositon of resident immune cells has also not drastically changed at 2 h post PDT compared with the non-treated tumour. Given the fact that the total cell yield was reduced by approximately half at 2 h after PDT, these results indicate that the level of killing of different types of resident leucocytes and malignant cells was similar.

*Cytotoxicity of TAMs after PDT*

The occurrence of a pronounced invasion of leucocytes into PDT-treated tumour may, together with the other inflammatory changes, affect the activity of immune cells directed against the tumour. To test this, we harvested TAMs contained in SCCVII tumour using a differential attachment technique employed in our earlier studies (Korbelik *et al.*, 1991). Staining with monoclonal antibodies showed that over 90% of the cells selected in this way were Mac1$^+$ and ~70% were F4/80$^+$. The cytotoxicity of these cells against *in vitro* cultured SCCVII malignant cells was examined. The results (Figure 6) show that TAMs harvested from a PDT treated SCCVII tumour at 2 h after the treatment were almost five times more effective in the cytolysis of target cells than the TAMs from non-treated tumour.

**Figure 6** Cytotoxicty of tumour-associated macrophages (TAMs), selected from PDT treated and non-treated SCCVII tumours, against *in vitro* cultivated SCCVII cells. The cultured (malignant) SCCVII cells were labelled with [$^3$H]thymidine and admixed with macrophages harvested from SCCVII tumours as described in the Materials and methods section. The treatment of Photofrin-based PDT of SCCVII tumours used for TAMs isolation was as in Figure 1, with the tumour excision performed at 2 h post PDT. The bars show s.d.

### Discussion

Dramatic changes occur in the cellular composition of SCCVII tumours treated with Photofrin-based PDT. The most striking change is the massive invasion of neutrophils into the tumour that starts rapidly (within 2 min) after the onset of photodynamic light treatment. Within 5 min, the neutrophil content in the treated tumour increased 200-fold, before decreasing once again to the levels seen in non-treated tumours between 2 and 4 h after PDT. Mast cells were the other type of immune cells that started to invade the tumour very early after the onset of photodynamic light treatment (within 5 min). The number of these cells in non-treated SCCVII tumour is very low (about 1000 cells in a 100 mg tumour) but their levels were more than five times higher between 0.5 and 4 h after PDT. Mast cells are powerful mediators of inflammatory response, and their recruitment, in spite of the low tumour content, could have a pronounced effect in the PDT-treated tumour.

Selective labelling of circulating leucocytes enabled the detection of the PDT-induced infiltration by another myeloid cell type into treated SCCVII tumours between 0 and 2 h post PDT. These cells expressed the Gr1 antigen (a common myeloid cell marker) and most of them stained negatively for the F4/80 antigen (a marker for mature macrophages). Based on this fact, and on the morphological examination, we concluded that the majority of these cells were most likely monocytes.

The percentage of malignant cells in SCCVII tumour decreased markedly during the photodynamic light treatment owing to the massive neutrophil invasion. The proportion of these cells in the tumour decreased even further during the next several hours following light delivery because of newly infiltrating monocytic cells. However, the total number of malignant cells in the tumour started to decrease substantially only after 2 h post PDT, presumably because of rapidly developing tumour tissue ischaemia (Henderson *et al.*, 1985).

The results based on the identification of resident malignant and immune cells in SCCVII tumour in the experiments with Hoechst suggest that within the first 2 h after PDT all these cells exhibit a similar sensitivity to the lethal effects of PDT. The only exception may be the inactivation of monocytes, suggested by the reduction in tumour content of 'other myeloid cells' observed at 0 h post PDT. A fast disappearance of newly infiltrated neutrophils from the treated tumour indicates that these cells were killed relatively quickly. These cells as well as 'other myeloid cells' might have been localised in the area of tumour vasculature already extensively damaged during the light treatment. In contrast, the percentage distribution of cellular populations at 4 h post PDT (pie graph in Figure 1) implies that newly infiltrated monocytes may outlive most of the tumour resident cells.

Taken together, the above results portray a typical scenario of acute inflammatory infiltration of myeloid cells at the affected site. The rapid and massive neutrophil invasion is promptly accompanied by the arrival of mast cells. Massive release of chemotactic substances from degranulating mast cells (Kerdel *et al.*, 1987) and dying neutrophils, as well as from damaged membranes of tumour cells (alluded to in the introduction), is the probable impelling force behind another wave of infiltration, this time involving monocytes, that follows 1–2 h later. There was no evidence for the participation of lymphocytes in these PDT-induced tumour infiltration events during the observation period of this study (up to 8 h post PDT).

The inflammatory reaction in PDT-treated tumour appears to have similarities with the cutaneous inflammation, identified as the main factor in PDT-induced skin phototoxicity, in which neutrophils and mast cells also have a critical role (Lim, 1989).

The data demonstrating a pronounced increase in the tumoricidal activity of TAMs (Figure 6) offer evidence that this inflammatory response is actually associated with the functional activation of immune cells. In a related study with cultured macrophages and tumour cells, we have demonstrated that *in vitro* PDT treatment of tumour cells (but not normal cells) potentiates their killing by macrophages (Korbelik and Krosl, 1994). The implication is that potentially reparable damage induced by PDT in tumour cells triggers macrophage-mediated tumoricidal activity. Such activity of non-specific immune cells may lead in a later phase to the development of a T-cell-specific immune activity. The ingestion of PDT-damaged or -killed tumour cells by macrophages, which are antigen-presenting cells, can result in tumour antigen presentation, with the consequent induction of tumour specific immunity (Yamamoto *et al.*, 1992). Such a development of PDT-induced immunopotentiation is suggested by the work of Canti *et al.* (1994). A number of reports have documented enhancement of tumour control by combining PDT with a variety of immunotherapy regimens

(Myers et al., 1989; Bellnier, 1991; Dougherty et al., 1992; Dima et al., 1994; Krosl and Korbelik, 1994). This supports the idea that PDT-induced immune reaction may be amplified and directed towards more effective tumour destruction.

Much work remains to be done in exploring the effects of PDT on cellular composition of tumours treated with different Photofrin/light dose combinations, examining the effects in different tumour models and with other photosensitisers. Each of these aspects requires a substantial experimental effort.

**Acknowledgements**

The authors wish to acknowledge technical assistance of Nancy LePard and Denise McDougal in flow cytometry analysis, and helpful advice from Drs DJ Chaplin and RE Durand. This study was supported by Grant MA-12165 from the Medical Research Council of Canada.

**References**

AGARWAL ML, LARKIN HE, ZAIDI SIA, MUKHTAR H AND OLEINICK N. (1993). Phospholipase activation triggers apoptosis in photosensitized mouse lymphoma cells. *Cancer Res.*, **53**, 5897-5902.

BELLNIER DA. (1991). Potentiation of photodynamic therapy in mice with recombinant human tumor necrosis factor-α. *J. Photochem. Photobiol. B, Biol.*, **8**, 203-210.

BELLNIER DA AND HENDERSON BW. (1992). Determinants of photodynamic tissue destruction. In *Photodynamic Therapy, Basic Principles and Clinical Applications*, Henderson BW and Dougherty TJ (eds.) pp. 117-127. Marcel Dekker: New York.

CANTI G, LATTUADA D, NICOLIN A, TARONI P, VALENTINI G AND CUBEDDU R. (1994). Antitumor immunity induced by photodynamic therapy with aluminium disulfonated phthalocyanines and laser light. *Anti-Cancer Drugs*, **5**, 443-447.

CHIEN KR, ABRAMS J, SERIONI A, MARTIN JT AND FARBER JK. (1978). Accelerated phospholipid degradation and association membrane dysfunction in irreversible ischemic liver cell injury. *J. Biol. Chem.*, **256**, 4809-4817.

DIMA VF, VASILIU V, LAKY D, IONESCU MD AND DIMA SV. (1994). Treatment of rat Walker-256 carcinosarcoma with photodynamic therapy and endotoxin irradiated with high energy electrons. *SPIE*, **2078**, 547-557.

DOUGHERTY GJ, ALLEN CA AND HOGG MH. (1986). Application of immunological techniques to the study of the tumour-host relationship. In *Handbook of Experimental Immunology*, Vol. 2, Weir DM, Herzenberg LA and Blackwell C. (eds.) pp. 125.1-125.12. Blackwell Scientific Publications: Oxford.

DOUGHERTY GJ, DOUGHERTY ST, KAY RJ, LANSDORP P AND HUMPHRIES RK. (1989). Identification and characterization of 114/A10, an antigen highly expressed on the surface of murine myeloid and erythroid progenitor cells and IL-3-dependent cell lines. *Exp. Hematol.*, **17**, 877-882.

DOUGHERTY GJ, THACKER JD, McBRIDE WH, KROSL G AND KORBELIK M. (1992). Effect of immunization with genetically-modified tumor cells on tumor recurrence following photodynamic therapy. *Lasers Med. Sci.*, **7**, 226.

FINGAR VH, WIEMAN TJ, WIEHLE SA AND CERRITO PB. (1992). The role of microvascular damage in photodynamic therapy: the effect of treatment on vessel constriction, permeability and leukocyte adhesion. *Cancer Res.*, **52**, 4914-4921.

HENDERSON BW AND DONOVAN JM. (1989). Release of prostaglandin $E_2$ from cells by photodynamic treatment *in vitro*. *Cancer Res.*, **49**, 6896-6900.

HENDERSON BW AND FINGAR VH. (1994). The role of vascular photodamage in photodynamic therapy. *Photochem. Photobiol.*, **59** (Suppl.), 1S-2S.

HENDERSON BW, WALDOW SM, MANG S, POTTER WR, MALONE PB AND DOUGHERTY TJ. (1985). Tumor destruction and kinetics of tumor cell death in two experimental mouse tumors following photodynamic therapy. *Cancer Res.*, **45**, 572-576.

HOWARD BV, MORRIS HP AND BAILEY JM. (1972). Ether-lipids, glycerol phosphate dehydrogenase and growth rate in tumors and cultured cells. *Cancer Res.*, **32**, 1533-1538.

KERDEL FA, SOTER NA AND LIM HW. (1987) *In vivo* mediator release and degranulation of mast cells in hematoporphyrin derivative-induced phototoxicity in mice. *J. Invest. Dermatol.*, **88**, 277-280.

KORBELIK M. (1993). Distribution of disulfonated and tetrasulfonated aluminium phthalocyanine between malignant and host cell populations of a murine fibrosarcoma. *J. Photochem. Photobiol. B, Biol.*, **20**, 173-181.

KORBELIK M AND KROSL G. (1994). Enhanced macrophage cytotoxicity against tumor cells treated with photodynamic therapy. *Photochem. Photobiol.*, **60**, 497-502.

KORBELIK M, KROSL G, OLIVE PL AND CHAPLIN DJ. (1991). Distribution of Photofrin between tumour cells and tumour associated macrophages. *Br. J. Cancer*, **64**, 508-512.

KROSL G AND KORBELIK M. (1994). Potentiation of photodynamic therapy by immunotherapy: the effect of Schizophyllan (SPG). *Cancer Lett.*, **84**, 43-49.

LIM HW. (1989). Role of mediators of inflammation and cells in porphyrin-induced phototoxicity. *SPIE*, **1065**, 28-33.

LYNCH DH, HADDAD S, KING VJ, OTT MJ, STRAIGHT RC AND JOLLES CJ. (1989). Systemic immunosuppression induced by photodynamic therapy (PDT) is adoptively transferred by macrophages. *Photochem. Photobiol.*, **49**, 453-458.

McBRIDE WH, THACKER JD, COMORA S, ECONOMOU J, KELLEY D, DUBINETT SM, HOGGE D AND DOUGHERTY GJ. (1992). Genetic modification of a murine fibrosarcoma to produce IL7 stimulates host cell infiltration and tumor immunity. *Cancer Res.*, **52**, 3931-3937.

MYERS RC, LAU BHS, KUNIHIRA DY, TORREY RR, WOOLLEY JL AND TOSK J. (1989). Modulation of hematoporphyrin derivative-sensitized phototherapy with *Corynebacterium parvum* in murine transitional cell carcinoma. *Urology*, **33**, 230-235.

NGWENYA BZ AND YAMAMOTO N. (1986). Effects of inflammation products on immune systems: lysophosphatidylcholine stimulates macrophages. *Cancer Immunol. Immunother.*, **21**, 174-182.

OLIVE PL, CHAPLIN DJ AND DURAND RE. (1985). Pharmacokinetics, binding and distribution of Hoechst 33342 in spheroids and murine tumours. *Br. J. Cancer*, **52**, 739-746.

PASS HI. (1993). Photodynamic therapy in oncology: mechanisms and clinical use. *J. Natl Cancer Inst.*, **85**, 443-456.

SNYDER F AND WOOD R. (1969). Alkyl alk-1-enyl-ethers of glycerol in lipids from normal and neoplastic tissues. *Cancer Res.*, **29**, 251-257.

THOMAS JP, HALL RD AND GIROTTI AW. (1987). Singlet oxygen intermediacy in the photodynamic action of membrane bound hematoporphyrin derivative. *Cancer Lett.*, **36**, 295-302.

YAMAMOTO N AND NGWENYA BZ. (1987). Activation of mouse peritoneal macrophages by lysophospholipids and ether derivatives of neutral lipids and phospholipids. *Cancer Res.*, **47**, 2008-2013.

YAMAMOTO N, ST CLAIRE DA, HOMMA S AND NGWENYA BZ. (1988). Activation of mouse macrophages by alkylglycerols, inflammation products of cancerous tissues. *Cancer Res.*, **48**, 6044-6049.

YAMAMOTO N, HOOBER JK, YAMAMOTO N AND YAMAMOTO S. (1992). Tumoricidal capacities of macrophages photodynamically activated with hematoporphyrin derivative. *Photochem. Photobiol.*, **56**, 245-250.

ZURIER RB. (1982). Prostaglandins and inflammation. In *Prostaglandins*, Lee JB. (ed.) pp. 91-112. Elsevier: New York.